# An Australian Example of Translating Psychological Research into Practice and Policy: Where We are and Where We Need to Go

**DOI:** 10.3389/fpsyg.2016.00200

**Published:** 2016-02-19

**Authors:** Aliza Werner-Seidler, Yael Perry, Helen Christensen

**Affiliations:** Black Dog Institute, University of New South WalesSydney, NSW, Australia

**Keywords:** translation, knowledge translation, mental health, practice, policy, implementation

## Abstract

Research findings from psychological science have identified interventions that will benefit human health. However, these findings are not often incorporated into practice-based settings or used to inform policy, in part, due to methodological and contextual limitations. A strategic approach is required if we are to find a way to facilitate the translation of these findings into areas that will offer genuine impact on health. There is an overwhelming focus on conducting more clinical trials, without consideration of how to ensure that findings from such trials make it to the patients or populations for whom they were intended. The aim of this paper is to outline how the Black Dog Institute, an Australian medical research institute, has created a framework designed to facilitate the translation of research findings into practice-based community settings, and how these findings can be used to inform policy. We propose that the core strategies adopted at the Black Dog Institute to prioritize and implement a translational program will be useful to institutes and organizations worldwide to augment the impact of their work. We provide several examples of how our research has been implemented in practice-based settings at a community-level, and how we have used research in psychology as a platform to inform policy change.

## The Need for a Strategic Approach to Translational Research

Translational research is often conceptualized as the translation from basic research to new clinical interventions that can be used to improve human health. This common notion of ‘bench-to-bedside’ translation overlooks the fact that most new, evidence-based interventions will disappear into the ether for an extended period before potentially being incorporated into routine clinical practice approximately 17 years later (Institute of Medicine, 2001). In fact, only 14% of evidence based interventions are believed to enter day-to-day clinical practice at all (Westfall et al., 2007). Despite extraordinary advances in the development of complex interventions, there has been relatively little attention directed toward the transfer of results from empirical research into health practice and policy (Westfall et al., 2007). One of the reasons for this is that much of the focus has been on moving basic research to the clinical trial stage using highly controlled methodology, without consideration of how to translate successful interventions into practice at a community level (Lenfant, 2003; Graham et al., 2006; Green, 2014). However, leading commentators and scientists are increasingly recognizing the need for ‘context-sensitive science’. This concept refers to the development of knowledge within the context in which is intended for use, which is achieved via transdisciplinary collaboration (Gibbons, 2000). It is suggested that by working across disciplines in the environment intended for eventual application, the knowledge that is developed will be robust and reliable in the targeted setting, rather than being limited to controlled laboratory conditions. The implication is that research with greater ecological validity will facilitate the integration of new knowledge into practice (Gibbons, 2000; Glasgow et al., 2006).

There has been some shift within the academic community and government funding bodies toward this model, with increased recognition of the need to close the gap between the emergence of a promising intervention and the availability of the treatment to consumers and policy makers (Kessler and Glasgow, 2011; National Health and Medical Research Council [NHMRC], 2012). For example, in some parts of the world (e.g., UK, Canada), research councils are now mandating that researchers strategically engage collaboratively with key stakeholders including policy makers and practitioners at the conception of a project, as a way to translate their findings (Darzi, 2007; Canadian Institutes of Health Research, 2012). This illustrates the first step toward prioritizing the translation of research findings into practice and policy domains. Leaders in the field have proposed that a fundamental change in attitudes toward scientific research that is more ecologically valid than the standard lab-based randomized controlled clinical trial (RCT) is needed, together with greater funding and scientific recognition of implementation science (Kessler and Glasgow, 2011; Perry and Bennett-Levy, 2014). That said, changes in research culture and procedures at the organizational level are also integral to building momentum and contributing to this paradigm shift (Graham et al., 2006).

The aim of the current paper is to outline how the Black Dog Institute (the Institute), a medical research institute operating independently as a not-for-profit organization, based in Sydney, Australia, has created a framework and set of strategies to facilitate the translation of research findings in mental health into community settings, and how these findings can be used to inform policy. We propose that this review of activities at the Black Dog Institute may serve as a small-scale example of how broader systemic change can occur. We propose that the core strategies adopted at the Institute to prioritize and implement a translational program will be useful to organizations worldwide to facilitate the translational impact of their work. As we will demonstrate, embedding a translational approach both within the Institute, and externally via partnering with organizations and stakeholders, is required if we are to prevent valuable research findings from being ‘lost in translation’. We will outline the specific steps the Institute has taken to achieve translational outcomes, including several examples of how our research lends itself to implementation in practice-based settings at a community-level, and how we have used research in psychology as a platform to inform policy change.

## Overview of Institute Structure

**FIGURE 1:**
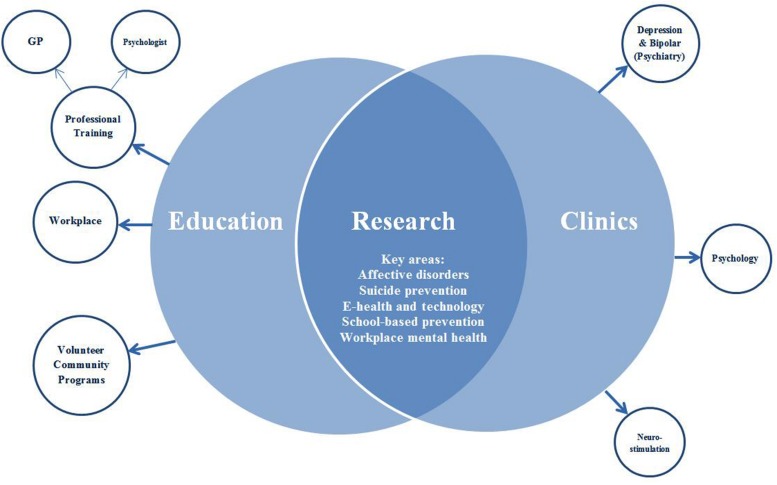
**Basic structure of the Black Dog Institute and its three pillars (represented as overlapping circles)**.

The Institute is an independent, non-government, university-affiliated not-for-profit organization. The mission of the Institute is to *‘enable mentally healthier lives through innovations in science, medicine, education, public policy and knowledge translation’*. The strategic approach to facilitate this mission is *‘the development and dissemination of knowledge needed to understand, prevent, and treat the significant mental health challenges facing the world’* (Black Dog Institute, 2015). Unlike the majority of mental health organizations, the Institute is unique in that it spans the translational continuum from basic research to clinical trials, community care, outpatient clinics, education, and policy development. The Institute involves key stakeholders and consumers at the outset of a project and for the duration of the project, to ensure that each stage is aligned with stakeholder interests (Graham et al., 2006). The three pillars of the Institute make this feasible: research, education, and clinical services (see **Figure 1**).

The structure of the Institute evolved organically, led by a team of psychiatric researcher-clinicians who recognized the need for community education programs and clinical services, embedded within a research institute. The leadership of the Institute recognized the added value of co-location, as a way of integrating research, clinical services, and education pillars.

The research team undertake original research spanning health promotion, illness prevention, early intervention, treatment, and recovery. The main research interests at the Institute are: online and mobile interventions for mental health disorders; prevention of mental health disorders; suicide prevention; neurostimulation; and workplace mental health. In 2014, there were 183 peer-reviewed publications produced by the Institute across these areas. The education team deliver programs to three distinct audience groups: to the community, to physicians and mental health professionals, and to workplace employers and employees. In 2014 alone, there were over 25,000 recipients of our educational programs. There is also a clinical services facility, with four clinics (depression, bipolar, psychology, and neurostimulation). These clinics are staffed by psychiatrists and psychologists who provided care to over 1400 individuals in 2014. The depression and bipolar clinics are staffed by psychiatrists, who offer a second-opinion psychiatric assessment service with recommendations and feedback provided to the referring professional, for clients with mood disorders. In contrast, the psychology clinic (staffed by clinical psychologists) offers ongoing psychological therapy for individuals with a range of presentations including stress, relationship problems, psychosis, and eating disorders, with a particular focus on anxiety and mood disorders, and associated suicidality. The neurostimulation clinic is a treatment clinic for individuals with depression, staffed by psychiatrists, offering neurostimulation assessments and transcranial magnetic stimulation treatment for depression. Referrals to clinics are made from either primary care service providers or private psychiatrists, and services are provided with partial support from the Australian Government’s Medicare scheme. Clients who are actively suicidal and in crisis are referred to a crisis management service (or hospital when indicated).

## Where We are: Translational Activities at the Black Dog Institute

The Institute has developed the infrastructure and in-house expertise across research, education and clinical services to allow it to conduct activities that span the full translational continuum from project development to delivery and uptake of evidence-based practices. Translational work is facilitated through the numerous overlapping projects across each pillar, cross-group collaboration, staff meetings, seminars, workshops, and social events. While this structure confers numerous advantages, there are several challenges in bringing together the three pillars to better meet our translational objectives. First, the issue of internal communication is critical to facilitate the exchange of information across the researchers, clinicians, educators, and support staff. We are currently introducing strategic opportunities to further facilitate communication between these groups, such as the introduction of a staff intranet and funding for cross-disciplinary collaborative projects. Planning is also underway to provide staff workshops that provide the terminology and understanding of how each pillar contributes to the translational program, which is expected to increase engagement.

### Development of the Translational Program

**FIGURE 2:**
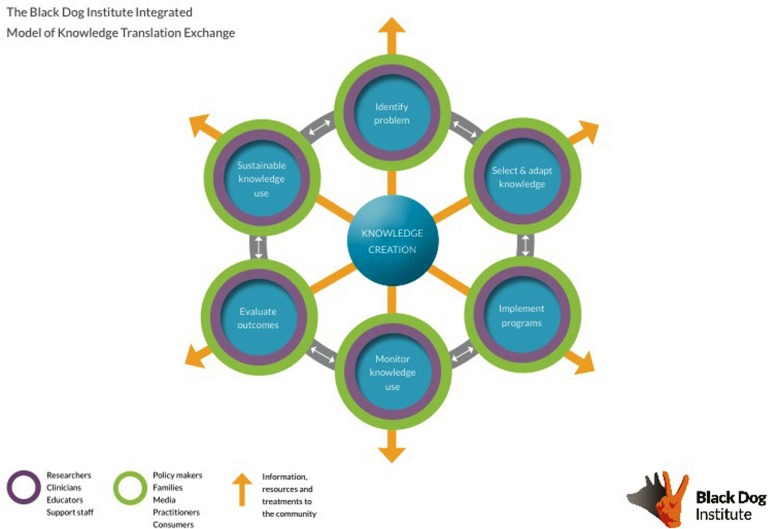
**The translational model at Black Dog Institute**.

The first step in developing a translational program at the Institute was the decision by the Executive to make translation a primary strategic priority. Making translation a key strategic direction of the Institute is supported by work showing that effective change within an organization must come from senior levels (Aarons et al., 2012). This aim was then defined through the development of an Institute-specific translational model, the purpose of which is to guide staff in recognizing how their role contributes to the translational program, and to create a framework for translation. The introduction of an organization-specific framework is necessary to prepare the organization for change, as well as to increase the likelihood that it will be implemented and maintained (Rycroft-Malone, 2004; Damschroder et al., 2009; Rycroft-Malone and Bucknall, 2010). First, several dominant models of translation were reviewed and key elements and processes pertinent to the Institute were noted for later incorporation into the Institute-specific model (Glasgow et al., 1999; Graham et al., 2006; Damschroder et al., 2009). Next, extensive consultation with stakeholders internal to the Institute was undertaken to audit current activities, and also focus on the translational potential and goals of each part of the Institute. This consultation phase spanned beyond the three pillars, and involved individuals from marketing, communications, fundraising, the creative team, and management (the enablers). A draft of the model was developed and presented to stakeholders for feedback. Several iterations followed until stakeholders were satisfied that their team and activities were appropriately represented and encapsulated within the model (see **Figure 2**). Importantly, this model is dynamic and iterations will be developed as the translational activities of the Institute change and progress. To further facilitate the delivery and implementation of this model, a dedicated translational position was introduced; a strategy that has been garnering increased support (Ward et al., 2009). There is currently a translation committee that meets regularly to ensure that the translational agenda remains a priority.

A number of specific changes have been implemented to facilitate change and a greater consideration of translational issues across each pillar of the Institute. These are considered, in turn, below.

### Research

#### Consideration of Translational Issues Prior to Project Commencement

Taking into account the recommendation for translational work to direct greater attention to issues of external validity (Green and Nasser, 2012), investigators are required to address practical issues including feasibility, cost, accessibility, and acceptability, and a plan for the next translational phase, *prior* to commencing their research. That is, researchers are required to apply to conduct research at the Institute, and approval is provided only when these issues are satisfactorily addressed. This ensures that researchers have insight into the potential of their findings for delivery across health care settings and in the community (Landsverk et al., 2012).

#### Stakeholder Consultation

Consultation with consumer stakeholders in the planning phase underpins our work, and is intended to increase the likelihood that programs will be acceptable to recipients and fit for delivery within the clinic, health services, or educational settings. This has been emphasized even further at the Institute, with the establishment of our Lived Experience Advisory Panel in 2015, comprising a team of individuals with a personal experience of a mental health disorder to provide guidance on our research. Drawing from the principles of participatory research design (Minkler and Wallerstein, 2008), the involvement of other relevant stakeholders (as determined by the nature of project) is also commonplace at the Institute, and plays a critical role in minimizing potential barriers and obstacles to our work. For example, we routinely consult with schools and school administrators (e.g., principals) in the planning of projects that fall within our school-based mental health research stream on issues of feasibility and acceptability.

#### Change in Research Direction

An emerging research direction of the Institute is the use of innovation and technology to address mental health problems. This is particularly relevant in the context of translation because online technologies and e-health initiatives show tremendous potential in enhancing the availability, deliverability, and cost-effectiveness of mental health interventions to the community (Andrews et al., 2010; Christensen and Hickie, 2010).

### Clinical Services

#### Providing Research Opportunities to Clients

Clinicians feed into the research cycle by providing the opportunity for clients to participate in research. For clients who are interested, a meeting is set up with a clinical research assistant to provide information about studies on offer. This research assistant is not linked to any specific research project, but acts as an intermediary between the clinician and researchers so that no undue pressure is placed on clients to participate. Since the introduction of this system in February 2014, 51% of clients coming through the clinic have consented to meet with the research assistant about potential research opportunities, and of that group, 61% were eligible to participate in a study and agreed to having their details passed on to the relevant research team.

#### Identifying Gaps in Available Clinical Services

Clinicians provide feedback to researchers if there is a need in the community that is not being met. For example, in line with the well-documented limited accessibility to services in rural areas (e.g., Australian Institute of Health and Welfare, 2005), clinicians have recognized the growing need for specialized psychiatric services in these areas. Clinicians at the Institute are working with researchers to develop an online, stepped-care clinic that will offer online treatment and tele-psychiatry consultations to individuals across Australia (including rural areas). Taking the form of a pragmatic study, this approach will be implemented and evaluated simultaneously, with clear and immediate benefits for real-world application (Patsopoulos, 2011). This process is greatly aided by having the clinic and research teams in the same building, as the current health care system does not lend itself to synergy between clinicians and researchers in this way.

### Education

#### Delivery of Community Programs

Community programs are delivered by trained individuals with a lived experience of a mental health disorder in civic settings (e.g., libraries, surf clubs) and in schools, to facilitate accessibility and maximize reach. The aim of our community programs is to increase mental health awareness, decrease stigma, and facilitate help-seeking in the community. The programs are currently being evaluated against these objectives in a subsample of individuals who access them, with outcome data expected in the coming months.

#### Professional Training Programs

The in-house education team fosters collaboration and synergy between researchers and real-world practitioners (e.g., teachers, general practitioners). The professional training workshops provide continued professional development to general practitioners (physicians), and allied health professionals, and involve content derived from the relevant literature and best-practice guidelines in areas of speciality and expertise at the Institute (e.g., mood disorders). The decision to educate professionals emerged from the belief that primary care offers a convenient point of intervention and is often the first point of contact for individuals with mental health problems. These programs are regularly evaluated against the standards set out by the national professional licensing bodies, with in-house data showing that professionals report increased knowledge and confidence following program completion.

#### Workplace Mental Health Training Programs

The workplace mental health training programs evolved from pioneering work conducted by a research team, now located at the Institute (Harvey et al., 2009; Henderson et al., 2011). This work is an example of direct translation from research to implementation and dissemination in the workplace environment. As a relatively recent addition to our suite of educational programs, the impact of our workplace mental health program is currently undergoing evaluation.

### Integration with e-Health

It is important to note that the translational activities in the education team are substantially augmented by the emergence of e-health initiatives. Several of our programs are delivered using new technologies (e.g., webinars, interactive forums), thereby removing some of the barriers to reach professionals practicing outside of the local area. This is in line with an emerging field of ‘technology enabled knowledge translation’, which refers to the use of technology to accelerate the integration of knowledge to practice (Ho, 2012). These programs are delivered not only in metropolitan centers, but also rural and remote areas where access to health services is often limited (Parslow and Jorm, 2000; Caldwell et al., 2004).

### Reaching the Community: The Role of Communications

The Institute has a specific engagement strategy that is designed to connect our work and message with the community. This strategy is delivered through our marketing team, with the help of our creative director. Specifically, the creative director is responsible for making our translational messages accessible and engaging to our target audiences. We also have a marketing team who are responsible for using the images and concepts developed by the creative director and disseminating them across multiple channels, including social media, to deliver our messages. Importantly, there is evidence to support our reach with these initiatives, with over 67,000 Facebook and 18,400 Twitter followers, and 1,683,000 unique views of our website during the period January – June 2015.

Although not directly related, we also conduct a range of fundraising activities that focus on health and wellbeing (e.g., fun runs, interstate cycle challenges) and provide community members the opportunity to get involved. The diversity in the channels through which we disseminate our messages, and the variety of ways we find to connect with and involve community members, provides a solid foundation of support for our work at the community level. This is in line with our strategic aim to engage the community throughout the translational process, as maintaining these links between the Institute and the community and consumers ensures that what we eventually deliver remains relevant and acceptable to those for whom it was intended.

## Translation from Research to the Community at Institute

### Example 1: *HeadStrong*

The purpose of *HeadStrong* is to promote help seeking behavior among adolescents, who are particularly vulnerable to the onset of mental health problems at this key developmental stage (Kessler et al., 2005). Fewer than 23% of Australian young people with a mental health disorder reports seeking help over the past 12 months (Australian Institute of Health and Welfare, 2011), and this program specifically targets known obstacles to help-seeking including stigma and poor mental health literacy (Gulliver et al., 2010).

*HeadStrong* was initially developed at the Institute (in partnership with the Inspire Foundation – a not-for-profit mental health organization) as a classroom-based educational resource for use by health and physical education (HPE) teachers for delivery to adolescents. *HeadStrong* comprises five modules that include ready-to-use classroom activities and teacher notes, and takes approximately 10 hours of class time in total.

An important aspect of *HeadStrong* that lends itself to later stage implementation and scalability is the fact that the program was developed so that each module links directly to curriculum learning outcomes. This means that all Australian schools can complete their curriculum requirements using this resource. That *HeadStrong* meets curriculum requirements has implications for acceptability to teachers, and feasibility for delivery within the existing school-system. Specifically, teachers who are already under pressure to meet their teaching requirements can integrate this material into their regular schedule and have it count toward their students learning outcomes. This makes the program attractive to teachers in terms of reducing preparation time and collating of resources, but also in fulfilling curriculum requirements.

After *HeadStrong* was developed and pilot tested, an RCT was conducted to evaluate the impact of the resource on students’ mental health literacy and stigmatizing attitudes, relative to regular HPE classes. In a sample of 380 students from 10 schools, results showed improved literacy and decreased stigma in both groups. However, these effects were significantly greater for students who received *HeadStrong*, supporting its use (Perry et al., 2014). It is important to draw attention to the fact that this program was trialed in the context for which it was intended – with clear implications for the feasibility of its delivery in the school environment. Following empirical validation using gold standard RCT methodology, there is a common perception among researchers that their work is done, and the publication of their results means that practitioners will integrate and use these findings in their work (Green, 2008). In reality, this is not the case. To prevent this, in the case of *HeadStrong*, we have disseminated the resources to educators across the country free-of-charge via a website created for this purpose. We have also conducted two national mail-out campaigns providing promotional materials and letters to every Australian high school (∼3500). Over the past 3 years, the resource has been uniquely downloaded 4212 times. Training resources, developed by the Institute education team, have also been made available to support the use of *HeadStrong*, through face-to-face workshops and online webinars. To date, 364 teachers from all seven Australian states and territories have participated in *HeadStrong* training workshops, and the *HeadStrong* Webinar Series has been accessed 2354 times. The original version of *HeadStrong* is currently being updated by adding additional modules. Importantly, this updated version will be publicized and disseminated nationally using strategic marketing and communication strategies, including holding online training sessions for teachers.

**FIGURE 3:**
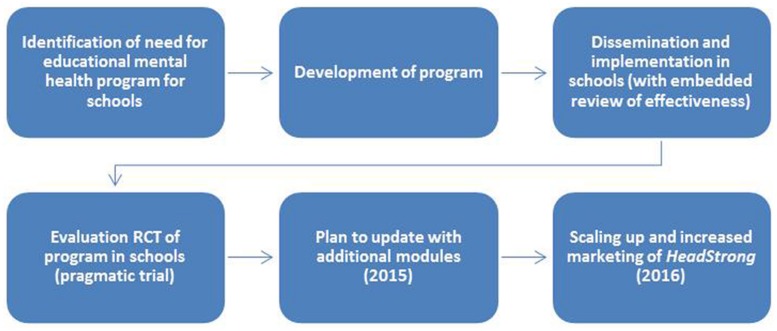
**Development, evaluation, implementation, and dissemination of *HeadStrong***.

*HeadStrong* provides a strong illustration of the full spectrum of translational work conducted at the Institute, from the synthesis of evidence to inform the development of a novel resource, to its empirical evaluation and implementation in a real-world setting, to dissemination and uptake into the community (see **Figure 3**).

### Example 2: A Systems Approach to Suicide Prevention

The second example of how the Institute is using evidence to change policy and improve health and community services takes a remarkably different approach to that described above. Building on existing evidence-based strategies to reduce suicide, we are proposing a systems approach to suicide prevention that requires the coordination of government, health services, and local community service providers to implement change across Australia. The problem of suicide is a global one, and there is accumulating evidence supporting the use of multiple components to reduce suicide, including reducing access to lethal means of suicide, responsible reporting by the media, gatekeeper training in schools and the military, training of general practitioners, improving public awareness, treatment for those with mental illness and appropriate and continuing care once people leave emergency departments (Mann et al., 2005; van der Feltz-Cornelis et al., 2011).

Research emerging from Europe has suggested that the best way to prevent suicide on a large scale is by taking a multi-level, multifactorial, systems-based approach. For example, in one study conducted in the UK, investigators used a longitudinal, pre-post design, to show that the implementation of specific suicide prevention strategies embedded within the healthcare system lowered suicide rates relative to areas where recommendations were not implemented (While et al., 2012). In other studies, integrated and systematic implementation of quality assurance procedures produce a remarkable reduction in suicide rates of up to 50% (Coffey, 2007; Hampton, 2010; Székely et al., 2013).

Building on this work, we have proposed a national systems approach to reduce suicide in Australia. Following a request by the National Mental Health Commission (NMHC), the Institute was commissioned to provide literature reviews and synthesize data to help inform its work. The NMHC provides independent advice to the Commonwealth Government on mental health reform. With a change in government, the Commission was then asked to provide a report to the new Government on health reform, including suicide prevention strategies. While the report was being compiled, and in association with Suicide Prevention Australia and its prevention alliance, a focused campaign was instigated to raise awareness, and gather support and funding for a systems approach to suicide prevention spanning all major players in policy making and mental health. A systems approach is a useful structure for gaining sector buy-in because it provides a role for all agencies, to align to the plan, and to be aligned with each other in the non-government organization (NGO) and health space. For example, this focus on suicide awareness and its solution included the following steps:

• **Government** – Meetings with Federal and State government ministers and incoming staff (e.g., the federal government Minister of Mental Health and their chief of staff, the new Minister for Health); mail-outs to all politicians.
• **Mental Health Commissions** – Approaches to the National and State Commissioners (including the New South Wales Mental Health Commission, and the National Mental Health Commission).
• **Suicide Prevention Australia** – Reporting and input into the strategy from Suicide Prevention Australia and its other agencies.
• **Academic conference presentations** – including the National Suicide Prevention Conference.
• **Community presentations –** targeting police, mental health nurses, allied health professionals, and medical professionals to increase awareness of suicide prevention strategies.
• **Social marketing campaigns –** to keep the Institute’s community supporters aware of the issues around suicide prevention; media stories to promote the Institute’s plan.

**FIGURE 4:**
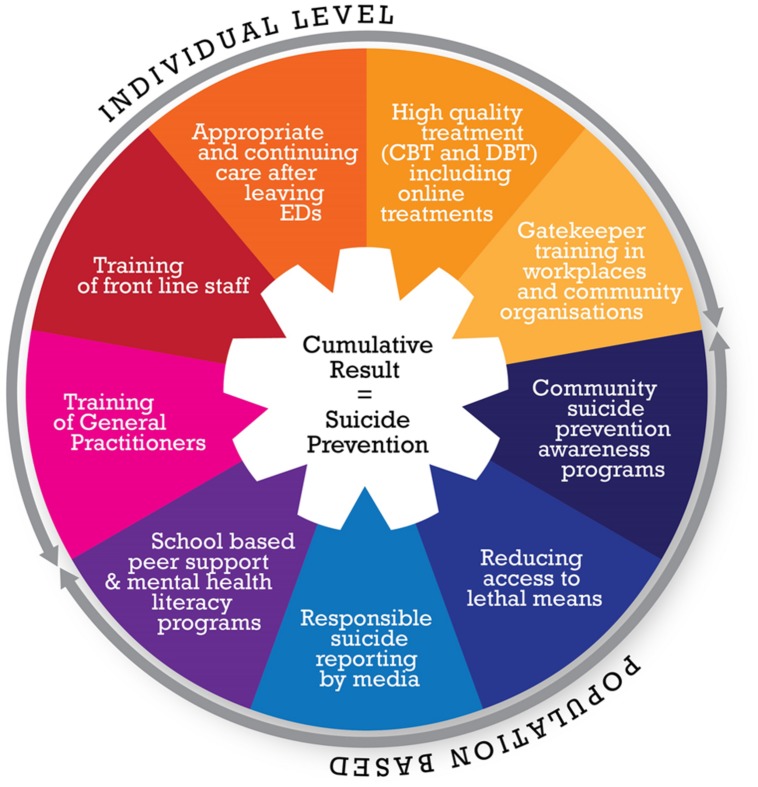
**Proposed suicide prevention strategies**.

The need for coordination between healthcare, community, and government levels is exemplified by the diversity of the nine strategies that we seek to implement (see **Figure 4**). It is the goal of this project to implement a national, systems-based approach that integrates empirically supported mental health services, community support, and education programs, and to evaluate whether these strategies can lower the suicide rate in Australia. The plan is for a phased, sequential roll out of the nine strategies across several regions to allow for the effects of the intervention to be determined over a 4-year period (similar to the design in the UK study). For the successful roll out of this initiative, each of the systems involved must work in concert for simultaneous implementation.

**FIGURE 5:**
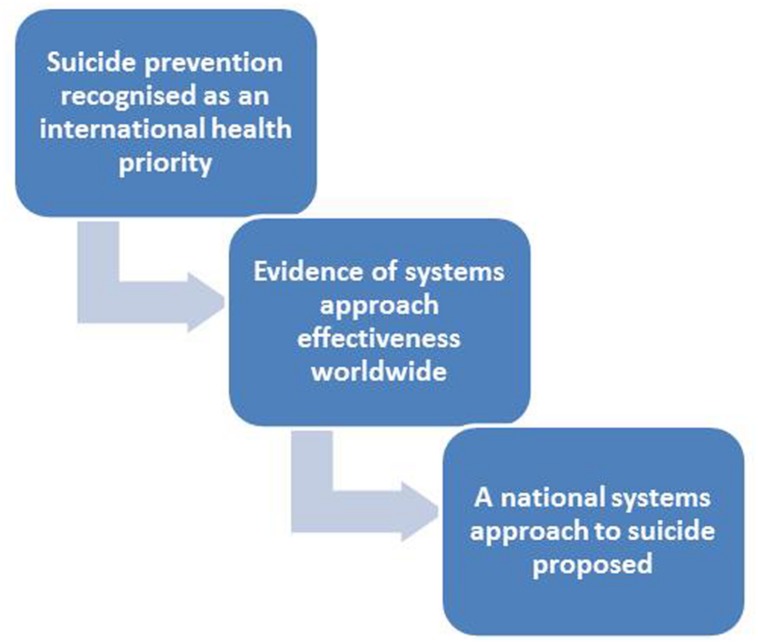
**Process of development of systems approach to suicide as the basis for national policy change in Australia**.

The systems approach to this suicide initiative is at an early stage where the feasibility of such an approach is being scoped, but illustrates how high quality research can be used to inform policy development (see **Figure 5**). The challenges are to bring all the thought leaders into alignment, and to underscore the importance of an evidence-based approach. Implementing an evidence-informed policy change and embedding the evaluation simultaneously protects against the possibility that an effective strategy or program (such as *HeadStrong*) might not get translated to the next stage and will become lost is the ‘quality chasm’ (Lenfant, 2003). All too often, policies are evaluated retrospectively, following their implementation. The simultaneous implementation and evaluation of a program ensures that if successful, the initiative can be easily implemented and scaled because issues of feasibility and effectiveness have already been considered.

## Where We Need to Go – Obstacles that Need to be Overcome in Using Psychology and Mental Health Research to Inform Practice and Policy

Two examples have been provided showing how research in mental health can be translated into practice-based settings and inform policy development. However, to maximize research translation for genuine impact, issues of funding, collaboration, sustainability, and commercialisation must be addressed.

### Resources Necessary

Perhaps the most considerable challenge in conducting a successful translational program at an organizational level is to secure the required funding and resources to support the aforementioned initiatives. In the case of the Institute, the financial resources of the Institute must first be contextualized in a landscape where in 2014, less than 10% of NHMRC government research funding was allocated to translational research, with the overwhelming majority (>70%) of funds allocated to highly controlled study designs that create original knowledge (NHMRC, 2014). Against this backdrop, the Institute relies on a diverse funding structure, with 35% of revenue coming from competitive grants awarded to researchers, and 25% provided by government health departments. The Institute generates income from in-house educational and clinical services (10%), but relies heavily on philanthropy and fundraising (20%) to operate. In a funding landscape of reduced government funding, philanthropic funding sources are becoming increasingly important. It may be worthwhile for other research settings to consider this as well. At the Institute, there is a dedicated team working to maintain relationships with key individuals and organizations who may wish to donate or invest in our work. This reflects recognition from senior management that securing funding from philanthropic sources has potential to ensure the sustainability of the Institute. Diversity in the funding structure creates a level of sustainability, but requires significant human, and financial resources.

### Collaboration

The structures surrounding the resourcing of the Institute mean that it is not possible (nor desirable) to conduct all aspects of translational research (from basic science to the implementation and dissemination phase) alone. An imperative is that organizational partnerships are fostered to allow us to implement, scale-up, and maintain the programs that we develop, evaluate, and deliver. Without links (and collaborations) to the settings in which we seek to implement and disseminate our programs, the Black Dog Institute would not be distinguishable from scientists working inside a University, and unlikely to reach the interface with practitioners, clinicians and consumers. Progress has already been made through our partnerships with schools, indigenous groups, and medical practices across Australia and these partnerships have been greatly aided by technological advancement. The online space has opened up a new avenue for promotion of mental health information, dissemination of knowledge, training and support. Moreover, it provides an unparalleled opportunity to reach rural and remote areas that previously have not been afforded the opportunity to capitalize on some of the mental health programs available.

### Sustainability and Commercialisation

A related challenge is the sustainability of our community programs and empirically validated mental health products (mobile apps and online programs). Until now, we have relied on funding from the Australian health departments on a needs-basis to support ongoing programs that have shown to be beneficial to the community, and cost-effective. However, on-going funding, including from government sources, is rarely guaranteed, and therefore, the issue of commercialisation of our mental health programs, and technology solutions is up for debate. The prospect of commercialisation introduces a new dimension to the work of researchers and underscores the need to partner with business developers and health economists in order to plan for the sustainability of interventions and programs that are being invented.

### Conclusion

At the Institute, we have recognized the importance of translation. By making this a priority, we have provided an opportunity for staff to develop knowledge and the vocabulary to discuss translation, and to appreciate how their role contributes to the broader context of improving human health. We suggest that it is now prudent for other organizations to follow suit. Drawing on our experience with translation at the Institute, we suggest several changes that need to occur within organizations. First, a framework that accounts for the organization’s structure, activities, and goals should be established. This is necessary to guide the organization broadly toward a translational focus, but also provides a basis for implementing specific changes. There are a multitude of models in the field (e.g., Damschroder et al., 2009) that can be adapted to fit the needs and idiosyncrasies of an organization, which will most likely be flexible and adapted to reflect the changing needs and goals of the organization as progress is made. Further, allocation of internal funding must reflect the importance placed on translation. Greater funding for translational activities provides the necessary resources to implement a translational framework at an organizational level. While this funding must ultimately come from major funding bodies or other philanthropic sources, organizations can make a strategic decision to make this a priority. Moreover, the importance of organizational partnerships cannot be emphasized enough – no single organization can possibly have the resources required to develop, evaluate, implement, disseminate, and sustain programs that improve human health. It is only through improved synergy and collaboration between researchers, clinicians, educators, technological scientists, and policy makers that progress in translating findings into human health benefits can occur. A change in the allocation of funding on a national and international scale will be necessary if there is to be a paradigm shift toward funding more ecologically valid study designs that offer the greatest potential for translational progress.

While change at the level of professional organizations is a necessary starting point for broader change, the field of psychological science also requires a change in culture to reflect the growing need to work across the translational continuum. We have several suggestions on how this might be achieved. First, investment in training for students and early career researchers to develop the skills to design and implement studies that lend themselves to translational progression is required. Undergraduate science students have long been required to take courses in the history and philosophy of science. A relatively straightforward way to train a new cohort of young researchers would be to introduce a component on the history of translation and the tremendous ‘gap’ that exists, together with issues that need to be considered in order to close this gap. Second, there is a need for continued and improved communication between researchers, practitioners, and the community. While historically, there have been tensions between researchers and practitioners in particular, it is necessary for greater cooperation and collaboration between these groups if progress is to be made.

Against this context of a slowly changing culture of health and psychological research, it is imperative that translation remains on the agenda. Seventeen years for evidence-based treatments to make their way into the public health and policy spheres is too long. It is intended that this review of strategies implemented by the Black Dog Institute to improve the way in which we plan, implement, and disseminate our work to effect genuine, real-world impact, will offer some suggestion for how other organizations might do the same.

## Author Contributions

AW-S and YP developed the idea and structure of the manuscript; AW-S drafted the paper and received comprehensive feedback from YP and HC. All authors contributed to the intellectual content, and approved the final version.

## Conflict of Interest Statement

The authors declare that the research was conducted in the absence of any commercial or financial relationships that could be construed as a potential conflict of interest.
